# Comparison of a person-centered pregnancy prevention question and One Key Question to assess postpartum contraceptive needs

**DOI:** 10.1016/j.contraception.2024.110465

**Published:** 2024-04-16

**Authors:** Jayme L. Congdon, Eric Vittinghoff, Christine Dehlendorf

**Affiliations:** a Department of Pediatrics and Philip R. Lee Institute for Health Policy Studies, University of California, San Francisco, CA, United States; b Department of Epidemiology and Biostatistics, University of California, San Francisco, CA, United States; c Department of Family and Community Medicine, University of California, San Francisco, CA, United States

**Keywords:** Contraception, Family planning, Health services research, Patient-centered care, Postpartum

## Abstract

**Objectives:**

To explore the relevance of pregnancy intention as a screen for contraceptive needs among postpartum individuals.

**Study design:**

We surveyed 234 postpartum individuals to assess the alignment between pregnancy intentions in the next year and current desire to prevent pregnancy.

**Results:**

Most individuals (87%) desired pregnancy prevention now, including 73% of individuals who desired or were ambivalent about pregnancy in the next year.

**Conclusion:**

A majority of individuals considering pregnancy in the next year desired pregnancy prevention now. Directly assessing current desire to prevent pregnancy may be more specific for contraceptive needs in postpartum individuals.

**Implications:**

Our ability to ensure that all individuals who want to prevent pregnancy have access to contraception depends on the use of effective screening questions. These findings prompt consideration of broader clinical implementation of screening for desire to prevent pregnancy in lieu of questions about pregnancy intention in the next year.

## 1. Introduction

Addressing postpartum contraceptive needs is a critical component of perinatal care that can help to optimize birth outcomes, including by preventing undesired pregnancies with short interpregnancy intervals [1]. A prominent approach to determining need for contraceptive care has been to assess pregnancy intention in the next year, including in postpartum individuals [2,3]. One Key Question (OKQ) is an increasingly utilized means of assessing pregnancy intention in the next year [4]. However, OKQ, or pregnancy intention screening more broadly, have not been found to have a consistently positive impact on patient experience, provision of reproductive health services, or health outcomes [5–9]. Additionally, since inquiring about pregnancy intentions in the next year does not directly assess current desire to prevent pregnancy, clinicians may need to ask follow-up questions to clarify ambivalence and current health care needs [10]. Further, pregnancy intention is a complex and dynamic personal decision affected by many contextual factors that may be beyond the scope of a medical visit [11]. Additionally, not everyone relates to the concept of pregnancy planning [12]. Importantly, individuals who are ambivalent about future pregnancy or are planning pregnancy may still desire to use contraception, which clinicians could potentially miss if solely using pregnancy intention to identify care needs [13]. Understanding the relationship between pregnancy intention in the next year and current desire to prevent pregnancy in the postpartum population can help inform effective and patient-centered interventions to optimize the delivery of postpartum contraceptive care. We therefore conducted a cross-sectional survey of postpartum individuals, asking both pregnancy intention in the next year and current desire to prevent pregnancy.

## 2. Methods

We surveyed 234 English or Spanish-speaking individuals who were 2–6 months postpartum. Recruitment and data collection occurred at three pediatric primary care clinics in the San Francisco Bay Area, California that serve socioeconomically, racially, and ethnically diverse populations. Data collection occurred during July 2019–October 2020. Before March 2020, we recruited and administered electronic surveys in-person at well-child visits. Recruitment after March 2020 occurred by phone due to COVID-19 restrictions. We called eligible individuals up to three times within a week of their infants’ well-child visit and verbally administered surveys. Participants received $25 gift cards. We obtained informed consent prior to study procedures. The Institutional Review Board at the University of California, San Francisco approved the study.

Sociodemographic variables included self-reported age, race and ethnicity, and insurance type, as detailed in a prior publication [14]. We assessed desire to prevent pregnancy with an item previously used in statewide surveys, “*Do you want to prevent pregnancy now?*” with five response choices: *Yes, I am already doing something to prevent pregnancy*; *Yes, I want to start preventing pregnancy*; *No, I don’t want to prevent pregnancy*; *I am unsure whether I want to prevent pregnancy*; and *This question does not apply to me* [15]. We measured pregnancy intention using OKQ: “*Would you like to become pregnant in the next year?*” with four response choices: *Yes*, *No*, *Unsure*, and *OK either way*. We assessed pregnancy acceptability using, “*How would you feel if you got pregnant in the next year*?” with six response choices: *Very upset*, *Somewhat upset*, *Not sure*, *I wouldn’t mind*, *Somewhat pleased*, and *Very pleased*.

We conducted descriptive analysis of sociodemographic variables. We categorized desire to prevent pregnancy now and pregnancy intention responses as Yes, Ambivalent, or No and cross-tabulated frequencies (*n*) and relative frequencies (%). We cross-tabulated pregnancy prevention now and pregnancy intention and computed the 95% confidence interval (CI) for the difference in proportions.

## 3. Results

Of 305 individuals contacted, 263 (86%) participated. We excluded 29 individuals whose response to a primary outcome was “does not apply” or missing, resulting in a sample size of 234. Excluded individuals did not differ from individuals included in the final analytic sample in terms of age, race, ethnicity, or insurance type. Participants included in the final analytic sample were on average 32 years (range 17–51 years) and self-identified as Asian 15%, Black 10%, Hispanic/Latina 37%, White 28%, or multiracial or other 10%. Insurance type was commercial 60%, public 38%, or uninsured 2%.

**Fig. 1.**
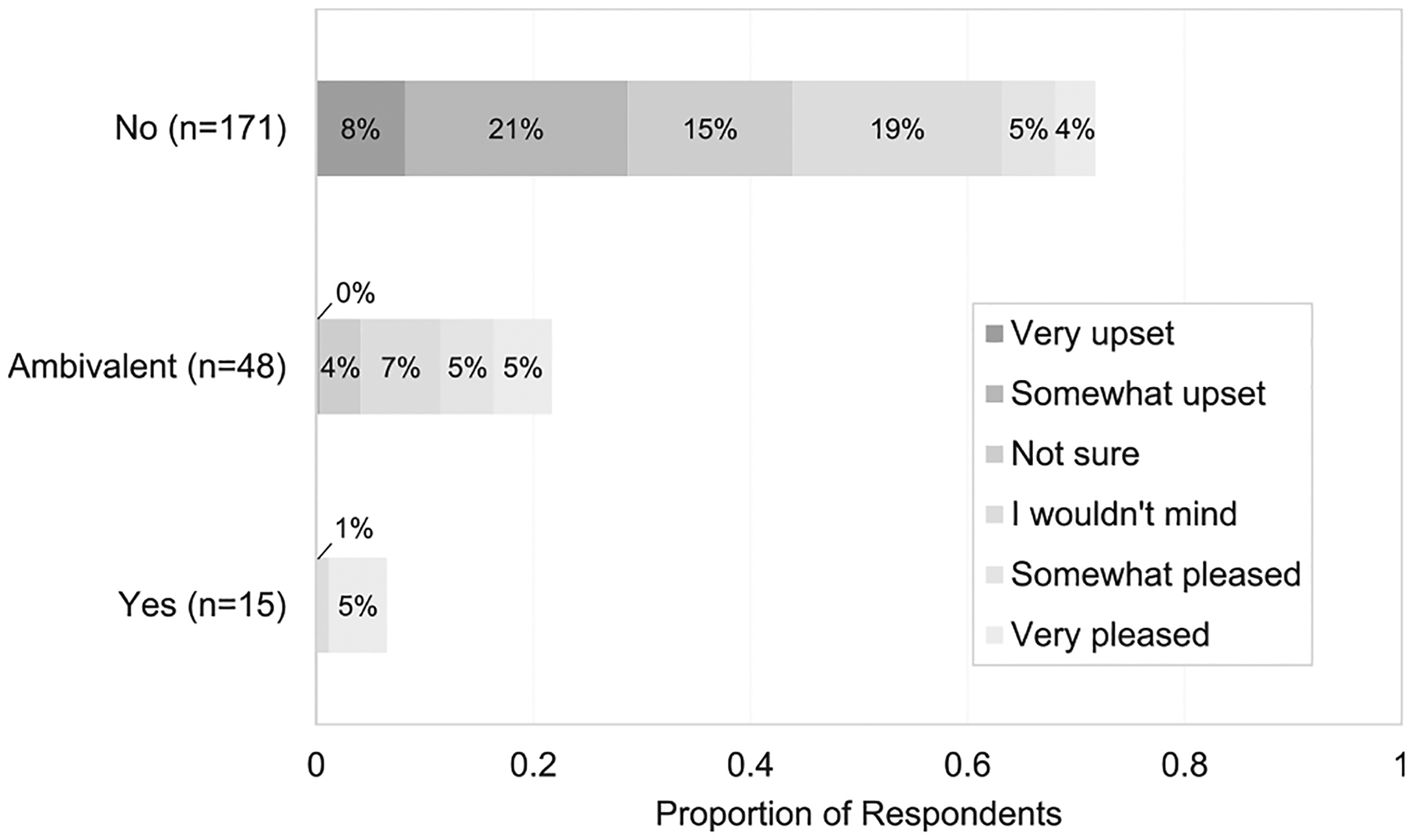
Pregnancy acceptability by pregnancy intention in the next year, among 2–6 months postpartum individuals in California 2019–2020 (*n* = 234). Pregnancy intention assessed using One Key Question, “*Would you like to become pregnant in the next year?*” with four response choices: *Yes*, *No*, *Unsure*, and *OK either way* (*Unsure* and *OK either way* were collapsed into an *Ambivalent* category); pregnancy acceptability assessed using *“How would you feel if you got pregnant in the next year?*”.

Most individuals (171 of 234; 73%) did not want to become pregnant in the next year, and 87% stated they wished to prevent pregnancy now – a 14% (95% CI 7, 21%) difference (Table 1). Of the 63 who wished to become pregnant in the next year or were ambivalent about pregnancy, 73% desired pregnancy prevention now, while among those not desiring pregnancy, 92% desired pregnancy prevention. The proportion of individuals with ambivalence about pregnancy in the next year was greater than the proportion with ambivalence about current desire to prevent pregnancy (21% vs. 5% – a 16% (95% CI 10, 22) difference. Pregnancy acceptability was highly variable among participants who were not planning a pregnancy or who were ambivalent (Fig. 1).

## 4. Discussion

In this study, almost three quarters of individuals who would consider pregnancy in the next year still desired pregnancy prevention. This finding demonstrates the potential limitation of pregnancy intention screening for identifying current pregnancy prevention needs. Determining current needs is particularly important given the benefit of preventing undesired, shortly spaced pregnancies [1], especially with the present lack of abortion access in much of the United States [16]. We found a high degree of ambivalence about pregnancy intentions and substantial variability in pregnancy acceptability across pregnancy intention groups, which aligns with literature on the complexity of pregnancy perceptions [17]. These results call into question the focus primarily on pregnancy intention by postpartum care providers aiming to identify current contraceptive needs. Furthermore, we posit that the clinician role should not be to resolve ambivalence about the personal decision to plan or to not plan a pregnancy, but rather to assess the current desire for pregnancy prevention. Shifting provider focus to patients’ current needs can generate more actionable clinical information for contraceptive counseling and is more consistent with diverse patient perspectives on family planning [12].

Pregnancy intention may have utility in public health and research contexts, however for clinical purposes, directly assessing current contraceptive needs is a more person-centered and directly actionable construct. This may be especially true during the postpartum months, which is a time of significant transition, when preferences about desired family size and birth spacing may evolve. Additionally, assessing current desire to prevent pregnancy may be more clinically expedient given the relatively low rate of ambivalence, compared to the more complex topic of pregnancy intention [18].

Our findings should be interpreted considering study limitations, including possible social desirability bias, e.g., overreporting desire to use contraception among women not intending pregnancy in response to normative expectations about pregnancy prevention. The context of declining pregnancy intentions during the early pandemic may also have affected survey responses, as well as what participants may have perceived as socially desirable responses [19]. Additionally, there may be limited generalizability to other populations or regions due to differences in the barriers and facilitators to accessing health care.

## Figures and Tables

**Table 1:** Cross-tabulation of pregnancy intention in next year (One Key Question) and current desire to prevent pregnancy, among 2–6 months postpartum individuals in California 2019–2020 (*n* = 234)

Pregnancy prevention now *“Do you want to prevent pregnancy now?”*	One Key Question: *“Would you like to become pregnant in the next year?”*			
	*Yes, want to become pregnant in next year*	*Ambivalent*	*No, do not want to become pregnant in next year*	Total
	*n*	*n*	*n*	*n*
	% (95% CI)	% (95% CI)	% (95% CI)	% (95% CI)
*Yes, want to prevent pregnancy now*	9 60% (35%−81%)	37 77% (63%−87%)	158 92% (87%−96%)	204 87% (82%−91%)
*Ambivalent*	1 7% (1%−35%)	4 8% (3%−20%)	6 4% (2%−8%)	11 5% (3%−8%)
*No, do not want to prevent pregnancy now*	5 33% (15%−60%)	7 15% (7%−28%)	7 4% (2%−8%)	19 8% (5%−12%)
Total	15 6% (4%−10%)	48 21% (16%−26%)	171 73% (67%−78%)	234

CI, confidence interval; OKQ, One Key Question.

Response choices for current pregnancy prevention (*Do you want to prevent pregnancy now?*) included: *Yes, I am already doing something to prevent pregnancy*; *Yes, I want to start preventing pregnancy*; *No, I don’t want to prevent pregnancy*; *I am unsure whether I want to prevent pregnancy* (labeled *Ambivalent* in the table); and *This question does not apply to me*. Response choices for OKQ (*Would you like to become pregnant in the next year?*) included: *Yes, No, Unsure,* and *OK either way* (*Ambivalent* defined as *Unsure* or *OK either way*).
